# Correction to *Lancet Infect Dis* 2020; 20: 1263–72

**DOI:** 10.1016/S1473-3099(21)00046-3

**Published:** 2021-03

*Meredith LW, Hamilton WL, Warne B, et al. Rapid implementation of SARS-CoV-2 sequencing to investigate cases of health-care associated COVID-19: a prospective genomic surveillance study.* Lancet Infect Dis *2020; **20:** 1263–72*—This Article should have been published under a CC BY Open Access license. The funding information has been corrected. These corrections have been made to the online version as of Jan 22, 2021.

